# Breakpoint junction features of seven DMD deletion mutations

**DOI:** 10.1038/s41439-019-0070-x

**Published:** 2019-08-22

**Authors:** Niall P. Keegan, Steve D. Wilton, Sue Fletcher

**Affiliations:** 1 Murdoch University, Perth, Australia; 2 Centre for Molecular Medicine and Innovative Therapeutics, Perth, Australia; 3 Perron Institute, Perth, Australia; 4 University of Western Australia, Perth, Australia

**Keywords:** Mutation, DNA sequencing, PCR-based techniques, Disease genetics

## Abstract

Duchenne muscular dystrophy is an inherited muscle wasting disease with severe symptoms and onset in early childhood. Duchenne muscular dystrophy is caused by loss-of-function mutations, most commonly deletions, within the *DMD* gene. Characterizing the junction points of large genomic deletions facilitates a more detailed model of the origins of these mutations and allows for a greater understanding of phenotypic variations associated with particular genotypes, potentially providing insights into the deletion mechanism. Here, we report sequencing of breakpoint junctions for seven patients with intragenic, whole-exon *DMD* deletions. Of the seven junction sequences identified, we found one instance of a “clean” break, three instances of microhomology (2–5 bp) at the junction site, and three complex rearrangements involving local sequences. Bioinformatics analysis of the upstream and downstream breakpoint regions revealed a possible role of short inverted repeats in the initiation of some of these deletion events.

## Duchenne muscular dystrophy: Understanding how the DMD gene breaks

Researchers in Australia have identified new examples of the genomic factors and mechanisms that lead to deletions linked with Duchenne muscular dystrophy (DMD). DMD is an inherited neuromuscular disease which causes progressive deterioration of muscles and, in some cases, intellectual impairment. Using samples from seven DMD patients, Niall Keegan of Murdoch University in Perth and colleagues sequenced the DNA left behind around the deletions in the *DMD* gene which cause the disease. They found one clean break, three sections with short repeated sequences, and three with more complex rearrangements. The diversity of these findings led them to suggest that the deletions resulted from a diversity of genomic factors and repair mechanisms. Future work could incorporate these findings into a model to predict where deletions will occur, expanding our understanding of DMD and its causes.

## Introduction

Duchenne muscular dystrophy is an inherited neuromuscular disease arising from loss-of-function mutations in the *DMD* gene. The primary symptom of Duchenne muscular dystrophy is a progressive deterioration of all muscles in the body apart from the extraocular muscles^1^. Mild to moderate intellectual impairment is also observed in some cases, and there is evidence of an inverse relationship between the severity of cognitive effect and the age of physical onset^2^. Intellectual impairment can be exacerbated in cases where the causative genomic lesion extends into nearby genes such as *NROB1* (ref. ^3^) and *GKD*^4^.

While a diverse array of mutation types can give rise to Duchenne muscular dystrophy or the symptomatically milder Becker muscular dystrophy, the majority of the pathological mutations of the *DMD* gene are deletions^5^, many of which encompass one or more entire exons.

The clearest predictor of the phenotype that will result from a large intragenic deletion is the set of exons lost. If the loss of these exons disrupts the gene’s open reading frame, the processed mRNA will either be translated into a truncated protein or be degraded by nonsense mediated decay before translation can be completed^6^. If, on the other hand, the deletion preserves the reading frame, it is far more likely that a functional protein isoform will be produced^7^, though this cannot be guaranteed^8^. While the *DMD* gene possesses a high degree of redundancy, especially in the central rod domain, not all *DMD* exons are equally dispensable^9^. An abridged dystrophin protein may retain much of its structural function, but loss of exons encoding the hinges^10,11^, actin-binding domain^12^, or dystroglycan-binding domain^13^ of the gene will necessarily impact the functions of those parts of the protein, leading to a phenotype distinct from that which may have arisen from a similarly sized in-frame deletion elsewhere in the gene.

The phenotype produced by a *DMD* whole-exon deletion is not predicted solely by the identities of the exons lost. Some intronic *DMD* sequences serve important regulatory roles both before^14,15^ and after mRNA splicing^16^, and deletions that affect functional regions such as these are likely to have negative consequences for the patient’s phenotype—consequences that can vary greatly even among patients with identical exon deletions^16^. In some cases, the unique conjunction of intronic sequences caused by a deletion can trigger the inclusion of a deleterious pseudoexon in the predominant transcript^17,18^. Given these factors and the role that intron secondary structure plays in pre-mRNA splicing^19^, it is plausible that the unique intronic span of a *DMD* deletion may also affect how readily the nearby exons will respond to antisense oligonucleotide-mediated exon-skipping therapy. For these reasons, it is of great value to both the patient and the researcher to fully map the intronic breakpoints in *DMD* whole-exon deletion cases, particularly those that manifest atypical phenotypes. Not only will this knowledge empower the patient to better understand their own disease, but comparison of symptoms between patients with fully mapped whole-exon deletions may lead to the discovery of new regulatory regions within the *DMD* introns and provide some insight into how these mutations occur.

In this study, successive rounds of PCR were used to delimit and amplify the genomic breakpoint junctions of seven Duchenne or Becker muscular dystrophy patients with whole-exon deletions. To economize on primers, we limited our study to deletions within the *DMD* deletion mutation hotspot, defined as spanning introns 44 to 55 (ref. ^20^). Bioinformatics web tools were then used to search the sequence around the breakpoints for features that may have contributed to each deletion event. Our analysis indicated that these deletions probably originated via multiple repair and recombination pathways.

## Materials and methods

### Selection of cell strains and DNA extraction

Seven patient myoblast or fibroblast cell strains were selected from our cell database (see Table 1). Each of the donor patients had been diagnosed with a deletion of at least one whole exon in the e45–e51 region of the *DMD* gene based on sequencing of their mRNA prior to the commencement of this study. We also selected a human myoblast strain not known to carry any disease-causing allele for use as a normal control. The eight cell strains were resurrected and cultured, and the DNA was extracted and purified using the PureLink Genomic DNA Kit from Invitrogen (ThermoFisher, Melbourne).

**Table 1:** Details of the seven *DMD* exon deletion cell strains used in this study

Patient ID	Exon deletion	ORF preserved?	Assigned phenotype	Cell type	Origin
d1	45–49	Yes	Becker muscular dystrophy	Myoblast	Dubowitz Neuromuscular Centre, London, United Kingdom
d2	45–47	Yes	Becker muscular dystrophy	Myoblast	Dubowitz Neuromuscular Centre, London, United Kingdom
d3	45–47	Yes	Becker muscular dystrophy	Myoblast	Dubowitz Neuromuscular Centre, London, United Kingdom
d4	45–50	No	Duchenne muscular dystrophy	Fibroblast	The Children’s Hospital at Westmead, Sydney, Australia
d5	46–51	No	Duchenne muscular dystrophy	Myoblast	Hammersmith Hospital, London, United Kingdom
d6	48–50	No	Duchenne muscular dystrophy	Myoblast	Dubowitz Neuromuscular Centre, London, United Kingdom
d7	51	No	Duchenne muscular dystrophy	Fibroblast	Dystrophy Annihilation Research Trust Centre, Bengaluru, India

### PCR

PCRs were performed using AmpliTaq™ Gold DNA Polymerase from Invitrogen (ThermoFisher, Melbourne). Sets of up to seven primer pairs were designed at intervals across each intron expected to bear a deletion breakpoint. These primer sets were used to perform multiplex PCRs of the target genomic DNAs, and the reaction products were visualized on an agarose gel. The number of bands obtained for each reaction indicated the approximate extent of the deletion, and this information was used to inform successively more focused rounds of primer design and multiplex PCR. Once the breakpoints on either side of each deletion had been sufficiently delimited, a final PCR across the junction generated an amplicon containing the junction sequence (see Table 2 for primer sequences).

**Table 2:** Sequences of primers used to amplify genomic deletion junctions (gene reference NG_012232.1(DMD_v001)) and corresponding amplicon regions

Patient ID	Forward primer (5′-3′)	Reverse primer (5′-3′)	Amplicon
d1	CATATGGTTTCTGGCCTTAG	TGCTGTGAACTACAAAGCAC	1,171,882–1,518,667
d2	GGAACAGTATTCTAGGCAGG	CATCCCTCCCTTCTATGAAC	1,265,936–1,449,576
d3	CACAAGGGTGTTAAGAACTACC	GATAGTTTCAATAATATGACCATG	1,329,623–1,453,916
d4	CACCTCTTCTCATCTAATTCC	CGATCACAATCTTCTGTGAAG	1,366,876–1,552,860
d5	CTATGAACAGGTATAAACCTG	CAGGACCAGCTTCTTGAACG	1,377,531–1,608,719
d6	GCCTATGGTAAGATTGGTTTC	CCCTTGAGAAATATCTCCAAC	1,427,297–1,563,083
d7	CTCCTATTTCAGCAAGTATC	GACCCTGGTAGGTACATCATG	1,548,853–1,584,826

### Sequencing

Junction amplicons were purified using Diffinity RapidTips™ (Chiral Technologies, Tokyo, Japan) and submitted to the Australian Genome Research Facility (Perth) for Sanger sequencing.

### Bioinformatics

As per Verdin et al.^21^, we defined the breakpoint regions as the 150 bp surrounding each deletion breakpoint in the reference sequence (UCSC Genome Browser assembly ID hg38, Chromosome X). In cases of breakpoint junction microhomology, the 3′ end of the homologous sequence defined the center of the upstream and downstream sequence.

The web utility *Non-B DB*^22^ was used to search the 150 bp surrounding each breakpoint for non-B DNA features (i.e., A-phased repeats, direct repeats and slipped motifs, G-quadruplex forming repeats, inverted repeats and cruciform motifs, mirror repeats and triplex motifs, Z-DNA motifs, and short tandem repeats). Sequences were entered in FASTA format, and the results were saved as text files.

The “Repeating Elements” track of the UCSC Genome Browser^23^ was used to search the breakpoint regions for transposable elements and other repetitive sequences, and screen captures were taken of any relevant features. The gnomAD genome database^24^ was also searched for previously reported common polymorphisms at the junction sites, which, if present, may have contributed to the initiation of the observed mutation.

## Results

**Fig. 1:**
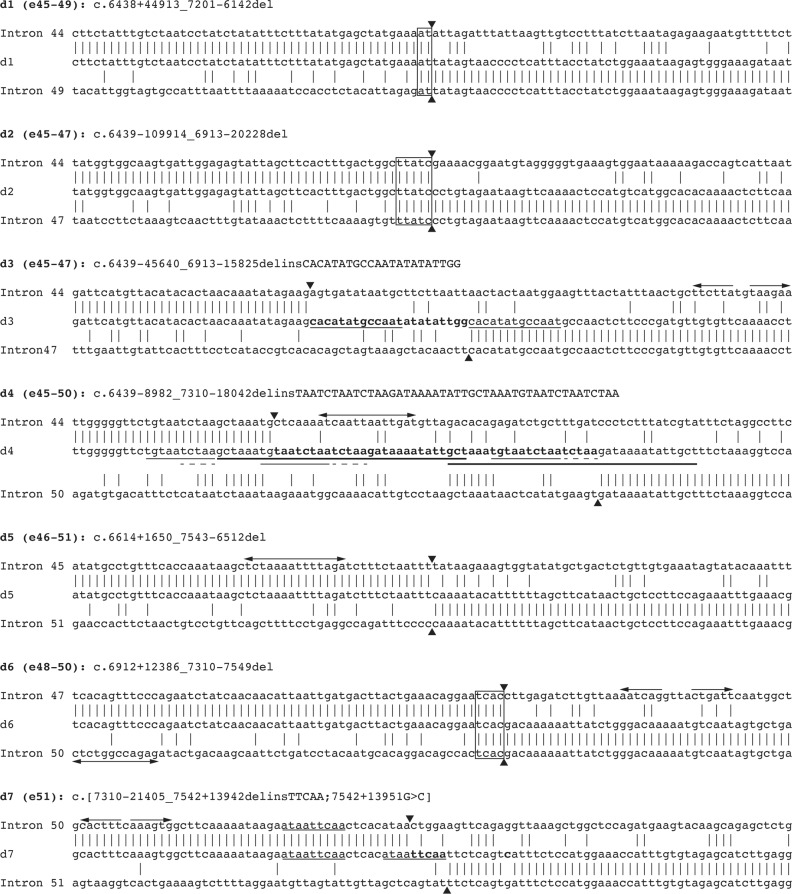
Junction sequences for seven patients with *DMD* whole-exon deletions, aligned to the corresponding regions of the reference sequence NG_012232.1(DMD_v001). Regions of microhomology are enclosed in boxes. Arrowheads indicate inferred breakpoint sites. New nucleotides are in bold type. Nonconsecutive direct repeats are underlined, with dashed and thicker lines used where necessary to distinguish complex repeat arrangements (d4). Left- and right-pointing arrows indicate the 5′ and 3′ spans, respectively, of short inverted repeats

Sequences of the deletion breakpoint regions for seven *DMD* patient cell lines are shown in Fig. 1, formatted in the style of Esposito et al.^25^. Microhomologies and other sequence anomalies are indicated where they occur, as are inverted repeats discovered in the corresponding regions of the reference sequence (NG_012232.1(DMD_v001)). Aside from the inverted repeats, no other non-B DNA structures were detected.

**Fig. 2:**
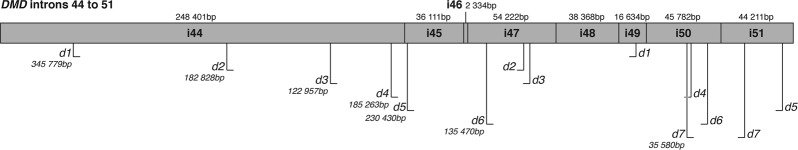
Relative positions of *DMD* gene deletion breakpoints for seven patients. Patient IDs are indicated, as are the total sizes of the deletions (excluding any de novo sequence inserted at the junction). Due to the large size of many of the introns in this region of the gene, intervening exons are not visible at this scale

The relative positions of each breakpoint within introns 44 to 51 of the *DMD* gene are shown in Fig. 2. The deletion junctions of patients d1, d2, and d6 exhibited microhomologies of 2, 5, and 4 bp, respectively. Patients d3, d4, and d7 each had inserted tracts of sequence at their deletion junctions. For patient d3, this inserted tract consisted of a 13 bp copy of the intron sequence immediately 3′ of the junction, followed by a 9 bp novel sequence (22 bp total). Patient d7’s junction showed a similar feature, though in this case, the inserted sequence is only 5 bp and appears to be a partial copy of a local 9 bp tract. A G > C substitution was also noted 9 bp 3′ of patient d7’s junction, though this is a commonly reported variant (rs6527115, dbSNP build 151—see ref. ^26^). The deletion junction of patient d4 exhibited a 45 bp de novo insertion that appeared to be composed of disordered and partially nested copies of tracts of the surrounding sequence. Patient d5’s deletion junction appeared to be a “clean break”, with no microhomology or de novo sequence insertions.

**Fig. 3:**
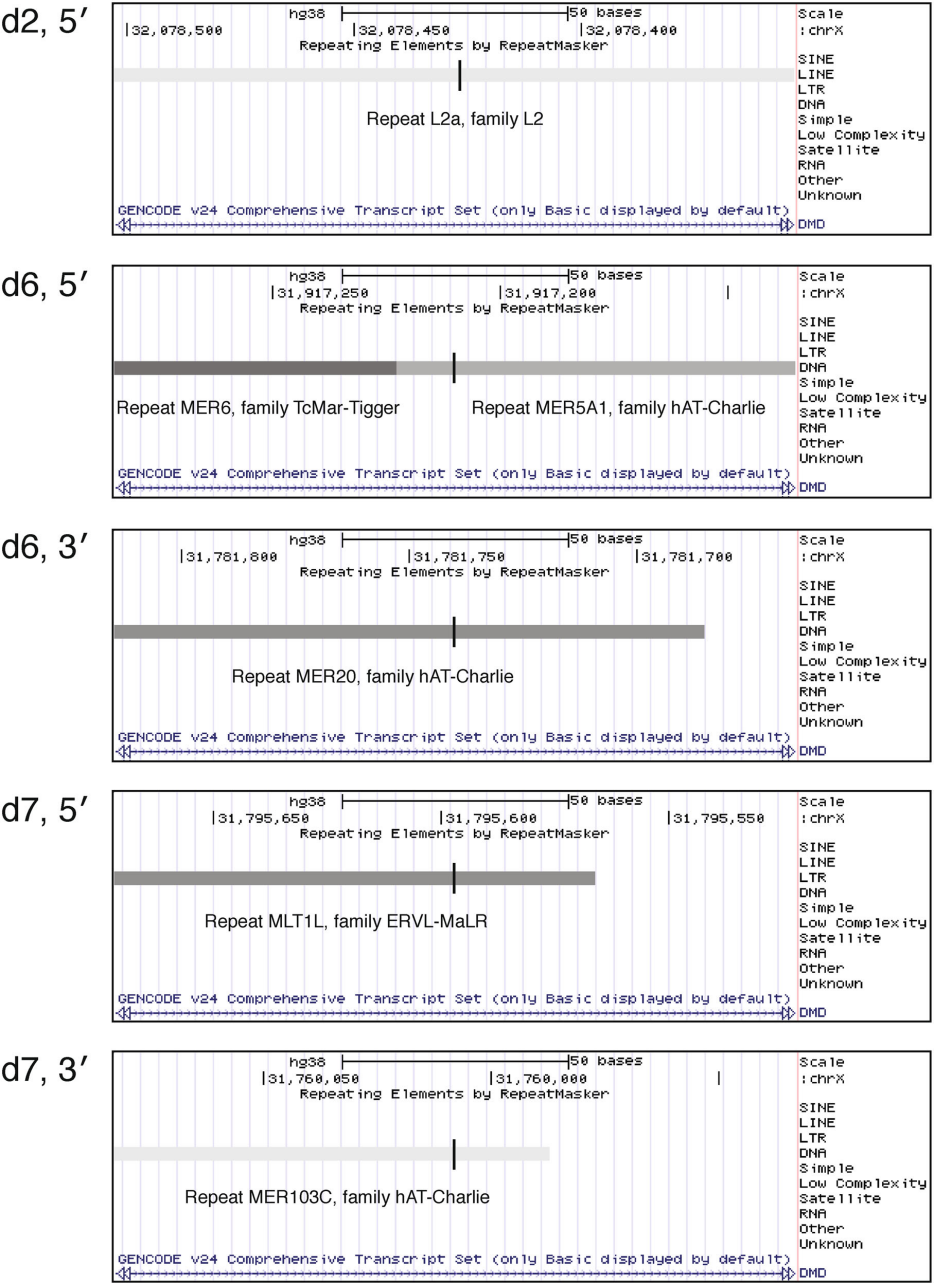
Repetitive elements detected within 150 bp deletion breakpoint regions of *DMD* exon deletion patients d2, d6, and d7. Vertical dashes indicate the breakpoint location relative to the normal sequence. Darker shading on a repetitive element indicates a stronger match to that element’s consensus sequence

The “Repetitive Elements” track of the UCSC Genome Browser found transposons and retrotransposons at 5 of the 14 reference sequence breakpoint regions. Images of these elements are shown in Fig. 3.

No previously reported common polymorphisms at the junction sites were found in the gnomAD database.

## Discussion

### Patients d1, d2, and d6—junction microhomologies

Deletion breakpoint junction microhomologies, such as those observed for patients d1, d2, and d6, are a feature common to many deletion mutations. In their 2013 study, Verdin et al.^21^ cataloged 22 microhomologies in the *FOX2* genes of separate patients, ranging in size from 1 to 66 bp. They found that microhomologies occurred at a much higher rate than would be expected if the upstream and downstream breakpoints were completely random (*p* = 2.28 × 10^–8^) and noted that the regions around the breakpoints tend to be significantly enriched with repetitive elements.

Patient d2’s deletion could be a product of microhomology-mediated end joining (MMEJ), repairing a double-stranded break in the *DMD*^27^. Other research has implicated MMEJ in large deletions of other genes^28^, and the microhomology observed in this case (TTATC) was within the 5–25 bp size range required for this repair pathway.

The breakpoints of the deletions of patients d1 and d6 (Fig. 1) were too small to be attributed to MMEJ (2 and 4 bp, respectively). It is possible that they arose from nonhomologous end joining (NHEJ), as use of this pathway can be assisted by small microhomologies at the breakpoints^29^ even though it does not strictly require them. Microhomology-mediated break induced replication (MMBIR) is also a possibility, as this pathway does not always create new sequences. However, this hypothesis is less likely, as evidence from other mammals indicates that MMBIR is responsible for substantially fewer breakpoint junctions than NHEJ^30^.

### Patients d3, d4, and d7—complex insertions at junctions

Patients d3, d4, and d7 all exhibited complex sequence insertions at their breakpoint junctions. A likely explanation for how these deletion junction sequences arose is MMBIR. This repair pathway is known to cause complex rearrangements of the DNA at the breakpoint junction, including duplications, inversions, and deletions^31^. MMBIR requires microhomologies at each breakpoint, and these were not observed for patients d3, d4, or d7. However, the microhomologies required for MMBIR need only be 1–3 bp long, and in cases where complex noncanonical junction sequences are created, such small sequence features could easily be obscured.

### Patient d5 deletion junction—clean break

The clean break observed at patient d5’s deletion junction indicates that this deletion probably arose via NHEJ^25^, as this is the only repair or recombination pathway known to be capable of producing clean break junctions.

### The role of transposable elements in facilitating large deletions

Transposable elements have been implicated as a causative factor in many large genomic deletions^32,33^, and it has been proposed that homology between two transposable elements facilitates non-allelic homologous recombination (NAHR)^34^. We detected a retrotransposon L2a site at the 5′ breakpoint region of patient d2, as well as transposons at both of patient d6’s breakpoints and both of patient d7’s breakpoints (Fig. 3). However, a BLAST-N analysis of each breakpoint pair for the deletions of patients d3, d4, and d7 could not detect any significant homology, and thus it does not appear that these sequence features contributed to the deletion events.

### The role of non-B DNA in large deletions

Non-B DNA conformations may facilitate large genomic deletions by interacting with local microhomologies to errantly initiate the double-stranded break repair mechanism^35,36^. Our analysis detected six inverted repeats (size range 12–16 bp) across six of the deletion breakpoint regions but did not find any examples of the other searched-for features.

Short inverted repeats have previously been implicated in the occurrence of large genomic deletions^37,38^ and Lu et al.^39^ found that inverted repeats as small as 7–30 bp are positively associated with deletion breakpoint locations in human cancers. While it is not possible to replicate Lu et al.’s statistical analysis with a sample size of 14 breakpoints from seven nonrandomly selected patient cell lines, our findings are at least compatible with the hypothesis that short inverted repeats play a role in the initiation of some of the studied deletion events.

## Conclusion

It has been suggested that large genomic deletion breakpoints do not occur randomly, but instead arise as a result of local features in the genome^21^. Indeed, there are a number of ways that asymmetrical chromosomal rearrangements can occur, producing deletions in tandem with other rearrangements, such as inversions or duplications^40^. Even with a sample size of just seven deletions from a single gene, we have observed a surprising diversity of junction phenomena and inferred a comparable diversity of contributing genomic factors and repair pathways. Developing a model that can incorporate all these factors and predict where and how deletions occur remains a daunting task—but it is a goal worth pursuing, both for its outcomes for human knowledge and for better informing genetic disease patients and their families.

## Data Availability

The reported nucleotide sequence data (d1–d7) are available in the GenBank database under accession numbers MK746139, MK829593, MK829594, MK829595, MK829596, MK829597, and MK829598.
